# Ultrasound‐induced changes in structural and physicochemical properties of β‐lactoglobulin

**DOI:** 10.1002/fsn3.646

**Published:** 2018-04-16

**Authors:** Shuang Ma, Xu Yang, Changhui Zhao, Mingruo Guo

**Affiliations:** ^1^ Department of Food Science College of Food Science and Engineering Jilin University Changchun China; ^2^ Department of Radiotherapy First Hospital of Jilin University Changchun China; ^3^ Department of Food Science Northeast Agriculture University Harbin China; ^4^ Department of Nutrition and Food Sciences College of Agriculture and Life Sciences University of Vermont Burlington VT USA

**Keywords:** β‐lactoglobulin, physicochemical properties, response surface methodology, ultrasound treatment

## Abstract

Effect of ultrasound treatment on the physicochemical properties and structure of β‐lactoglobulin were investigated. β‐Lactoglobulin was treated with ultrasound at different amplitudes, temperatures, and durations. The surface hydrophobicity and free sulfhydryl group of β‐lactoglobulin were significantly increased after ultrasound treatment (*p *<* *.05). The maximal surface hydrophobicity and free sulfhydryl group were 5,812.08 and 5.97 μmol/g, respectively. Ultrasound treatment changed the physicochemical properties of β‐lactoglobulin including particle size (from 1.21 ± 0.05 nm to 1.66 ± 0.03 nm), absolute zeta potential (from 15.47 ± 1.60 mV to 27.63 ± 3.30 mV), and solubility (from 84.66% to 95.17%). Ultrasound treatment increased α‐helix and β‐sheet structures of β‐lactoglobulin. Intrinsic fluorescence intensity of ultrasound‐treated β‐lactoglobulin was increased with shift of λ_max_ from 334 to 329 nm. UV absorption of β‐lactoglobulin was decreased with shift of λ_max_ from 288 to 285 nm after ultrasound treatment. There were no significant changes in high‐performance liquid chromatography and protein electrophoretic patterns. These findings indicated that ultrasound treatment had high potential in modifying the physiochemical and structural properties of β‐lactoglobulin for industrial applications.

## 1 INTRODUCTION

Bovine β‐lactoglobulin (~50%–55%) is the main fraction in whey proteins (Çelebioğlu, Gudjónsdóttir, Chronakis, & Seunghwan, 2016). It is a globular protein with 162 amino acids and a single polypeptide chain, including two disulfide bonds and one free sulfhydryl group at Cys121 (Papiz et al., 1986). Because of its distinct secondary and tertiary structures, it can be used in either native state or in form of thermally aggregated particles. β‐Lactoglobulin is a valuable ingredient in food manufacturing due to its high content of essential amino acids and versatility in terms of functional properties, such as gelling ability, emulsifying ability, and foaming ability (Bals & Kulozik, 2003). Dombrowski, Johler, Warncke, and Kulozik (2016) reported that β‐lactoglobulin is widely applied to provide stability of foam structures for its pronounced surface activity.

High‐intensity ultrasound (HIU), a type of nonthermal technique, has attracted great interest due to its promising effects in improving the quality and safety of processed foods (Ashokkumar et al., 2008; Frydenberg, Hammershoj, Andersen, & Wiking, 2013; Knorr, Zenker, Heinz, & Lee, 2004). Ultrasound (in general, >20 kHz) has become a widely used technique with many advantages in several dairy applications (Chandrapala, Oliyer, Kentish, & Ashokkumar, 2012; Chandrapala, Zisu, Kentish, & Ashokkumar, 2012), including improving the solubility and foaming properties of whey proteins (Arzeni et al., 2012; Jambrak, Lelas, Mason, Kresic, & Badanjak, 2009; Shen, Fang, Gao, & Guo, 2017) and altering the physical properties of gels made from milk (Zisu, Bhaskaracharya, Kentish, & Ashokkumar, 2010). HIU can cause some degree of protein unfolding and aggregation (Ashokkumar et al., 2009). Partial unfolding of the protein molecule and the exposure of hydrophobic groups induced by ultrasound increased the hydrophobic interactions and enhanced its foam‐forming ability. β‐Lactoglobulin with ultrasound treatment could significantly improve its functional properties, modify secondary structure, and lead to increase in surface hydrophobicity (Stanic‐Vucinic et al., 2012). Ultrasound treatment resulted in minimal disruption to the structure of β‐lactoglobulin but greater change to α‐lactalbumin (Chandrapala, Oliver, Kentish, & Ashokkumar, 2013). In our previous work, we observed that ultrasound treatment can significantly improve the structure and antioxidant activity of β‐lactoglobulin (Shuang, Cuina, & Mingruo, 2018). Therefore, further studies for the effect of ultrasound treatment on β‐lactoglobulin would be of great significance.

The objectives of this study were to investigate the effects of high‐intensity ultrasound treatment on physicochemical properties and structure of β‐lactoglobulin using response surface methodology. Changes in physicochemical properties of β‐lactoglobulin were analyzed including surface hydrophobicity, free sulphydryl group content, particle size, zeta potential, and solubility. Changes in structure of β‐lactoglobulin were analyzed by various spectroscopic techniques, including high‐performance liquid chromatography (HPLC), Fourier transform infrared (FT‐IR), intrinsic fluorescence, and UV spectroscopy. By doing this way, we attempted to find out a better way of improving β‐lactoglobulin's properties using ultrasound treatment.

## 2 MATERIALS AND METHODS

### 2.1 Materials

Raw bovine milk (nonfat solids ≥8.10%, protein 2.90%) was purchased from ChunGuang Dairy Co. LTD (Changchun, China). β‐Lactoglobulin (≥90%, lyophilized powder), 5,5′‐dithiobis‐(2‐nitrobenzoic acid) (DTNB, ≥98%, BioReagent, suitable for determination of sulfhydryl groups), and 8‐anilino‐1‐naphthalenesulfonic acid (ANS, ≥97.0%, for fluorescence) were from Sigma‐Aldrich (St. Louis, MO, USA). Acetonitrile was purchased from Fisher Corporation (HPLC grade, USA); trifluoroacetic acid was from Aladdin Corporation (HPLC grade, TFA, China). SDS‐PAGE loading buffer was from TaKaRa Biotechnology Co., Ltd. (Japan). BCA protein assay kit was from Beyotime Biotechnology (China). All other reagents were of analytical grade and supplied from Beijing Chemical Works (Beijing, China). A Milli‐Q deionization–reverse osmosis system (Millipore Corp., Bedford, MA, USA) was used to provide deionized water by filtering with a 0.22‐μm filter.

### 2.2 Preparation of β‐lactoglobulin

β‐Lactoglobulin was separated from raw bovine milk according to the method of Aschaffenburg et al. with some modifications (Aschaffenburg & Drewry, 1957; Neyestani, Djalali, & Pezeshki, 2003). During the isolation process, the obtained filtrate was centrifuged (3,000 × *g* at 4°C for 30 min) and the separated β‐Lactoglobulin was obtained by filtration through Whatman No. 4 filter paper. After dialysis, β‐lactoglobulin was obtained by freeze‐drying at 0.034 atm for 48 hr. Purity of β‐lactoglobulin was analyzed by HPLC.

### 2.3 Preparation of β‐lactoglobulin solution

β‐Lactoglobulin solutions were prepared by dispersing proper amount of β‐lactoglobulin powder in deionized water to 1% (ω*/v*) and then stirred (2,000 rpm) for 1 hr at room temperature. Then, its pH was adjusted to 7.0 with NaOH solution (2 M) and stored at 4°C overnight. All solutions were filtered through a syringe filter (0.45 μm) and equilibrated at room temperature before ultrasound treatment.

### 2.4 Ultrasound treatment

An Ultrasonic Processor (VCX 800, Vibra Cell, Sonics, USA) with a 13‐mm high‐grade titanium alloy probe (amplitude, 114 μm) threaded to a 3‐mm tapered microtip was used to sonicate 15 ml β‐lactoglobulin solutions in centrifuge tubes. All samples (1%, ω*/v*) were treated with ultrasound (20 kHz) and at the intensity of 60 W/cm^2^ for 10, 20, and 30 min (10 s: 5 s work/rest cycles, varying amplitude (20%, 30%, and 40%), and different temperatures (40, 45, and 50°C), and immersed in water bath to counteract the heat generated by ultrasound treatment. The probe was placed at the same distance from the base of liquid level for all ultrasound treatment.

### 2.5 Experimental design

On the basis of the single‐factor experiments, three independent variables—temperature (40–50°C), time (0–30 min), and amplitude (20%–40%)—were applied in this study to determine the response pattern through a Box–Behnken Design (BBD). The three variables of *X*_1_ *, X*_2_, and *X*_3_ were the coded variables for temperature, time, and amplitude, respectively, while the response values were the surface hydrophobicity and free sulfhydryl group. The mathematics model for optimization of dependent variables was based on the following equation:

(1) $$Y=\beta_{0}+\sum_{j=1}^{k}\beta_{j}X_{j}+\sum_{j=1}^{k}\beta_{\mathrm{jj}}X_{j}^{2}+\sum \sum_{\mathrm{ipj}}^{k}\beta_{\mathrm{ij}}X_{i}X_{j}$$

where *Y* is the observed response value predicted by the model; β_0_, β_*j*_, β_*jj*_, and β_*ij*_ are the regression coefficients for intercept, linearity, square, and interaction effect, respectively, *X*_*i*_ *, X*_*j*_ are independent coded variables (Neter, Wasserman, & Kutner, 1990).

The goodness of the model fit was evaluated by the coefficient *R* ^2^. The whole experimental design, data analysis, and quadratic model building were accomplished using the Design‐Expert Software (Trial Version 7.0.0, Stat‐Ease Inc., Minneapolis, MN, USA).

### 2.6 Determination of surface hydrophobicity

Surface hydrophobicity of β‐lactoglobulin was determined using 1‐anilino‐8‐naphthalenesulfonate (ANS) (8.0 mmol/L in phosphate buffer 0.01 mol/L, pH 7.0) as the fluorescence probe according to the method developed by Kato and Nakai (1980) with modifications. Each sample was diluted to five concentrations from 0.005 to 0.025 mg/ml using the same buffer. Each dilution was poured into a quartz cuvette, and the fluorescence intensity was measured at 25°C using a spectrofluorometer (RF‐5301PC, Shimadzu UV, Japan) at 390 nm (excitation wavelength, slit 5 nm) and 470 nm (emission wavelength, slit 5 nm), and the scanning speed was 10 nm/s. Surface hydrophobicity was calculated from the initial slope of the fluorescence intensity versus protein concentration plot of the serial dilutions as an index of surface hydrophobicity (H_0_).

### 2.7 Determination of free sulfhydryl group (–SH)

The surface free SH content of β‐lactoglobulin was determined using Ellman's reagent DTNB with some modifications (Shimada & Cheftel, 1988). Ellman's reagent was prepared by dissolving 0.2 g DTNB in 50 ml Tris‐glycine buffer (dissolved 10.4 g of Tris, 1.2 g of EDTA, and 6.9 g of glycine in deionized Milli‐Q water to 1 L, pH 8.0). β‐Lactoglobulin (1%, 0.5 ml) solution was diluted with 5 ml urea buffer (dissolved 10.4 g of Tris, 1.2 g of EDTA, 6.9 g of glycine, and 480 g of urea in deionized Milli‐Q water to 1 L, pH 8.0) and 20 μl of Ellman's reagent (4 mg/ml DTNB in Tris‐glycine buffer). The solution was then incubated for 15 min at room temperature and measured at 412 nm by a UV–Vis spectrophotometer (UV2550, Shimadzu, Tokyo, Japan). Free sulfhydryl group content was calculated by following formula:

(2) $$-\text{SH}\;\left(\mu \text{mol}/g\right)=\frac{73.53\times D\times A_{412}}{C}$$

where *D* is dilute factor of samples; *A*_412_ is the absorbance at 412 nm; *C* is protein concentration of samples (mg/ml).

### 2.8 Determination of particle size

The particle size of β‐lactoglobulin solution was measured by dynamic light scattering (DLS) using a Zetasizer Nano ZS 90 (Malvern Instruments, UK). A volume of 1 ml of β‐lactoglobulin solution (1%, ω*/v*) was transferred into a measuring cell, and the temperature was set at 25°C. All measurements were conducted in triplicate, consisting of 11 individual runs for 10 s and equilibration for 120 s. The detection was conducted at a scattering angle of 173°. The particle size and polydispersity index (PDI) were calculated based on the Stokes–Einstein equation as shown below:

(3) $$D=\frac{k_{B}T}{3\pi \eta d(h)}$$

where *D* is the diffusion constant; *k*_B_ is Boltzmann's constant; *T* is the absolute temperature; η is the dynamic viscosity; *d (h)* is hydrodynamic diameter.

(4) $$\text{PDI}=\frac{\sigma^{2}}{Z_{D}^{2}}$$

where PDI is the relative change; *Polydispersity* is the standard deviation; σ is the width; *% Polydispersity (% Pd)* is the variation coefficient, equals to PDI^0.5^ × 100.

### 2.9 Determination of zeta potential

Zeta potentials of β‐lactoglobulin solution were measured with a Zetasizer Nano ZS 90. All measurements were performed in triplicate and presented as mean ± *SD*. The zeta potential was calculated by the electrophoretic mobility based on the Henry equation as shown below:

(5) $$U_{E}=\frac{2\varepsilon zf(ka)}{3\eta }$$

where *U*_E_ is the electrophoretic mobility; ε is the permittivity; *z* is the zeta potential; *f* (*ka*) is Henry function, equals to 1.5 based on the Smoluchowski approximation; where *k* is the Debye length (nm^−1^), and α is the particle radius (nm); η is the dispersion viscosity (mPa s) (Pyell, Jalil, Pfeiffer, Pelaz, & Parak, 2015).

### 2.10 Determination of solubility

The solubility of β‐lactoglobulin was measured at pH 7 according to the method with some modifications (Shen, Shao, & Guo, 2016). All samples were lyophilized by freeze‐drying at 0.034 mbar for 24 hr (Christ, Alpha 1‐2 LDplus, Germany). The protein powder obtained was dispersed (1%, ω*/v*) in deionized Milli‐Q water. The samples were stirred for 30 min and equilibrated at room temperature for 1 hr. The concentration of β‐lactoglobulin was determined using BCAprotein assay kit.

### 2.11 High‐performance liquid chromatography

High‐performance liquid chromatography (HPLC) was performed on a reversed‐phase analytical column C_8_ (Sepax Bio‐C_8_, Sepax Technologies) with a silica‐based packing (5 μm, 300 Å, 4.6 × 250 mm, LC‐20A, Shimadzu, Japan).

Chromatographic conditions: Gradient elution was carried out with a mixture of two solvents: Solvent A consisted of 0.1% trifluoroacetic acid (TFA) in acetonitrile; and solvent B was 0.1% trifluoroacetic acid (TFA) in deionized water. The elution was carried out using a linear gradient of solvent according to the method of Bonfatti, Grigoletto, Cecchinato, Gallo, and Carnier (2008) with some modifications. Separations performed with the following program: linear gradient from 33% to 45% A in 35 min and return linearly to the starting condition in 1 min. Before sample injection, the column was re‐equilibrated under the starting condition of 33% for 8 min. Therefore, the total analysis time per sample was about 44 min. The injection volume was 10 μl, and the flow rate was 0.5 ml/min. The cell temperature was kept at 40°C, and the detection was made at a wavelength of 214 nm while the detection wavelength was from 190 to 800 nm. In addition, the slit width was 1.2 nm, the lamp setting was D2&W, and the column pressure was less than 18 MPa.

### 2.12 Sodium dodecyl sulfate–polyacrylamide gel electrophoresis

Sodium dodecyl sulfate–polyacrylamide gel electrophoresis (SDS‐PAGE) was performed using a Mini‐Protean Tetra Electrophoresis System (Bio‐Rad, USA). Fifteen microlitre of β‐lactoglobulin solution (1%, ω*/v*) was mixed with 5 μl 4× loading buffer (TaKaRa) and placed in thermostatically controlled (100°C) water bath for 3 min. Electrophoresis was run at 120 V for 80 min. After electrophoresis, the gels were stained for approximately 4 hr and destained for approximately 8 hr. The molecular weight standards ranged from 10 kDa to 180 kDa. The gels were analyzed using Image Scanner (Gel Doc XR+, Bio‐Rad, USA).

### 2.13 Fourier Transform Infrared (FT‐IR) spectroscopy

The β‐lactoglobulin samples were analyzed using a Perkin–Elmer Spectrum 100 FT‐IR Spectrometer (IR PRESTIGE‐21, Shimadzu, Japan). The FT‐IR spectra were recorded with 45 scans at 4 cm^−1^ resolution from 4,000 to 400 cm^−1^. KBr was dried at 150°C for 4 hr, and the KBr spectrum was recorded as background. KBr sample pellets were prepared by mixing of 1 mg of β‐lactoglobulin sample with 200 mg of KBr.

### 2.14 Intrinsic fluorescence spectroscopy

All samples were diluted to the concentration of 0.01 mg/ml with phosphate buffer (10 mmol/L, pH 7.0). Each dilution was poured into a quartz cuvette, and the fluorescence intensity was measured at 25°C using a spectrofluorometer (RF‐5301 PC, Shimadzu UV, Japan) at 280 nm (excitation wavelength, slit 5 nm) and 470 nm (emission wavelength, slit 5 nm). The scanning speed was 10 nm/s.

### 2.15 Ultraviolet (UV) spectroscopy

All samples were analyzed using a UV–Vis Spectrophotometer (UV‐2550, Shimadzu, Japan). The measurement was conducted with β‐lactoglobulin samples of 0.05% (ω*/v*) at 25°C. The UV spectrum scanning range was recorded from 200 to 600 nm, the sampling interval was 1.0 nm, the slit width was 2 nm, and the scan rate was set as high speed. Each scan was performed three times.

### 2.16 Statistical analysis

All measurements were performed in triplicate. The significant differences of data among samples were calculated using SPSS version 11.5 (SPSS Inc. Chicago). Data were checked for homogeneity by Leveneǐs test. One‐way analysis of variance (ANOVA) and then a least‐squared differences (LSD) model were applied when the data were homogeneous. Dunnett's test was used when the data were heterogeneous. All the figures were plotted by origin 8.0 (OriginLab Corporation, Northampton, USA). All the data were presented as mean ± standard deviation (*SD*). Differences were considered as significant when *p *<* *.05 at 95% level of confidence.

## 3 RESULTS AND DISCUSSION

### 3.1 Effect of temperature, time, and amplitude on the surface hydrophobicity and free sulfhydryl group

**Figure 1:**
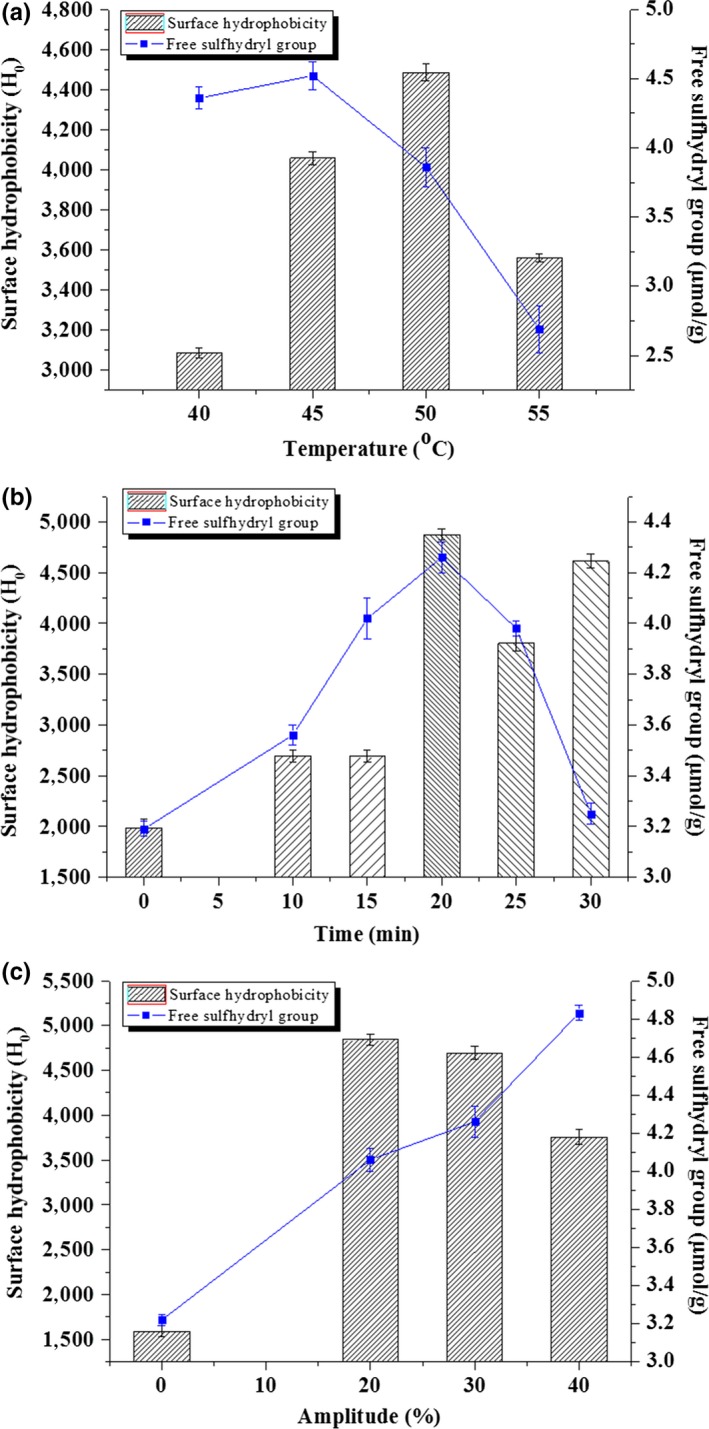
Effect of three factors on the surface hydrophobicity and free sulfhydryl group of β‐lactoglobulin

The effect of temperature, time, and amplitude on the surface hydrophobicity and free sulfhydryl group of β‐lactoglobulin was shown in Figure 1. The surface hydrophobicity of β‐lactoglobulin increased significantly when the temperature changed from 40 to 55°C. The maximum surface hydrophobicity was 4,484.1 at 50°C, but decreased remarkably when the temperature was above 50°C (Figure 1a). With the increase in temperature, the free sulfhydryl group of β‐lactoglobulin first increased and then decreased. The maximum free sulfhydryl group content was 4.52 μmol/g at 45°C. β‐Lactoglobulin may be denatured when the temperature was above 50°C.

The surface hydrophobicity and free sulfhydryl group of β‐lactoglobulin first increased and then decreased with the increase in time (Figure 1b). At 20 min, the maximum surface hydrophobicity and free sulfhydryl group content were 4,874.7 and 4.26 μmol/g, respectively. With the extension of ultrasonic time, ultrasound treatment changed the structural conformation of β‐lactoglobulin and induced changes in surface hydrophobicity and free sulfhydryl group.

The surface hydrophobicity first increased and then decreased with the increase in amplitude (Figure 1c). The maximum surface hydrophobicity was 4,841.8 at amplitude of 20%, while the free sulfhydryl group content increased with the increase in amplitude. High ultrasonic amplitude could increase acoustic energy and change the physicochemical properties of β‐lactoglobulin. Based on the results, the factors of temperature (40–50°C), time (10–30 min), and amplitude (20%–40%) were selected for Box–Behnken design.

### 3.2 Model fitting and response surface analysis

The experimental design and the response of surface hydrophobicity and free sulfhydryl group obtained for each experiment along with the predicted values are listed in Table 1. The adjusted *R* ^2^ and predicted *R* ^2^ were calculated to check the adequacy of the model. The significance of the model was calculated with analysis of *F*‐ratio and *p*‐value.

**Table 1:** Box–Behnken design and its responses

Experiments	*X*_1_	*X*_2_	*X*_3_	Response			
	Temperature (°C)	Time (min)	Amplitude (%)	Surface hydrophobicity		Free sulfhydryl group (μmol/g)	
				Experimental	Predicted	Experimental	Predicted
1	+1 (50)	+1 (30)	0 (30)	1,736	1,864.28	2.5	2.53
2	+1 (50)	−1 (10)	0 (30)	1,857.17	1,957.23	3.68	3.74
3	+1 (50)	0 (20)	−1 (20)	5,188.2	4,986.53	4.54	4.46
4	+1 (50)	0 (20)	+1 (40)	1,986.6	1,959.94	2.03	2.02
5	−1 (40)	0 (20)	+1 (40)	1,837.4	2,039.07	2.93	3.01
6	−1 (40)	0 (20)	−1 (20)	3,897.2	3,923.86	3.87	3.88
7	−1 (40)	−1 (10)	0 (30)	1,711.4	1,583.12	3.75	3.72
8	−1 (40)	+1 (30)	0 (30)	1,354.9	1,254.85	3.04	2.98
9	0 (45)	+1 (30)	−1 (20)	3,135.8	3,209.19	4.39	4.44
10	0 (45)	+1 (30)	+1 (40)	1,326.02	1,224.41	1.42	1.40
11	0 (45)	−1 (10)	+1 (40)	1,037.5	964.109	3.82	3.77
12	0 (45)	−1 (10)	−1 (20)	3,789.1	3,890.71	4.01	4.03
13	0 (45)	0 (20)	0 (30)	5,812.08	5,774.40	5.97	5.88
14	0 (45)	0 (20)	0 (30)	5,807.89	5,774.40	5.81	5.88
15	0 (45)	0 (20)	0 (30)	5,703.24	5,774.40	5.85	5.88

The mathematical model, representing the effect of three factors on the response values of surface hydrophobicity, can be described by the following quadratic equations (6) with analysis of variance shown in Table 2.

(6) $$\begin{array}{cc} Y_{1}=\; & 5,774.40+245.88X_{1}-105.31X_{2}-1,227.85X_{3}+58.83X_{1}X_{2}\\ & -285.45X_{1}X_{3}+235.46X_{2}X_{3}-1,602.15X_{1}^{2}-2,507.39X_{2}^{2}\\ & -944.91X_{3}^{2} \end{array}$$

**Table 2:** Variance analysis of surface hydrophobicity

Source	Sum of squares	*df*	Mean square	*F*‐value	*p*‐Value Prob > *F*
Model	45,369,059.76	9	5,041,006.64	144.26	<.0001
*X*_1_‐Temperature	483,670.55	1	483,670.55	13.84	.0137
*X*_2_‐Time	88,715.25	1	88,715.25	2.54	.1720
*X*_3_‐Amplitude	12,060,875.87	1	12,060,875.87	345.14	<.0001
*X*_1_ *X*_2_	13,845.05	1	13,845.05	0.40	.5567
*X*_1_ *X*_3_	325,926.81	1	325,926.81	9.33	.0283
*X*_2_ *X*_*3*_	221,756.23	1	221,756.23	6.35	.0532
*X*_1_^2^	9,477,673.61	1	9,477,673.61	271.22	<.0001
*X*_2_^2^	23,213,563.21	1	23,213,563.21	664.30	<.0001
*X*_3_^2^	3,296,680.51	1	3,296,680.51	94.34	.0002
Residual	174,723.09	5	34,944.62		
Lack of fit	167,117.98	3	55,705.99	14.65	.0646
Pure error	7,605.11	2	3,802.55		
Cor total	4,554,3782.85	14			

Comment: *R* ^2^ = .9962, Adjusted *R* ^2^ = .9893, Predicted *R* ^2^ = .9409, Adequate Precision = 31.52.

*Y*_1_ is the surface hydrophobicity, *X*_1_ *, X*_2_, and *X*_3_ are the coded variables for temperature, time, and amplitude, respectively. In general, the validity of the model can be judged by lack of fit to check the adequacy of a fitted‐response surface model (Liyana‐Pathirana & Shahidi, 2005). Table 2 shows that the *p*‐value of the response model was significant (*p *≤* *.0001), but the lack of fit was insignificant (*p *=* *.0646 > .05), indicating that the model could be used to analyze and predict the response of surface hydrophobicity. The *R* ^2^‐value was .9962, which was in good agreement with the Adj *R* ^2^‐value of .9893, and the Pred *R* ^2^‐value of .9409 was in reasonable agreement with the Adj *R* ^2^‐value of .9893 (Daneshvand, Ara, & Raofie, 2012). The *X*_1_ *, X*_3_ *, X*_1_ *X*_3_ were significant in model terms.

**Figure 2:**
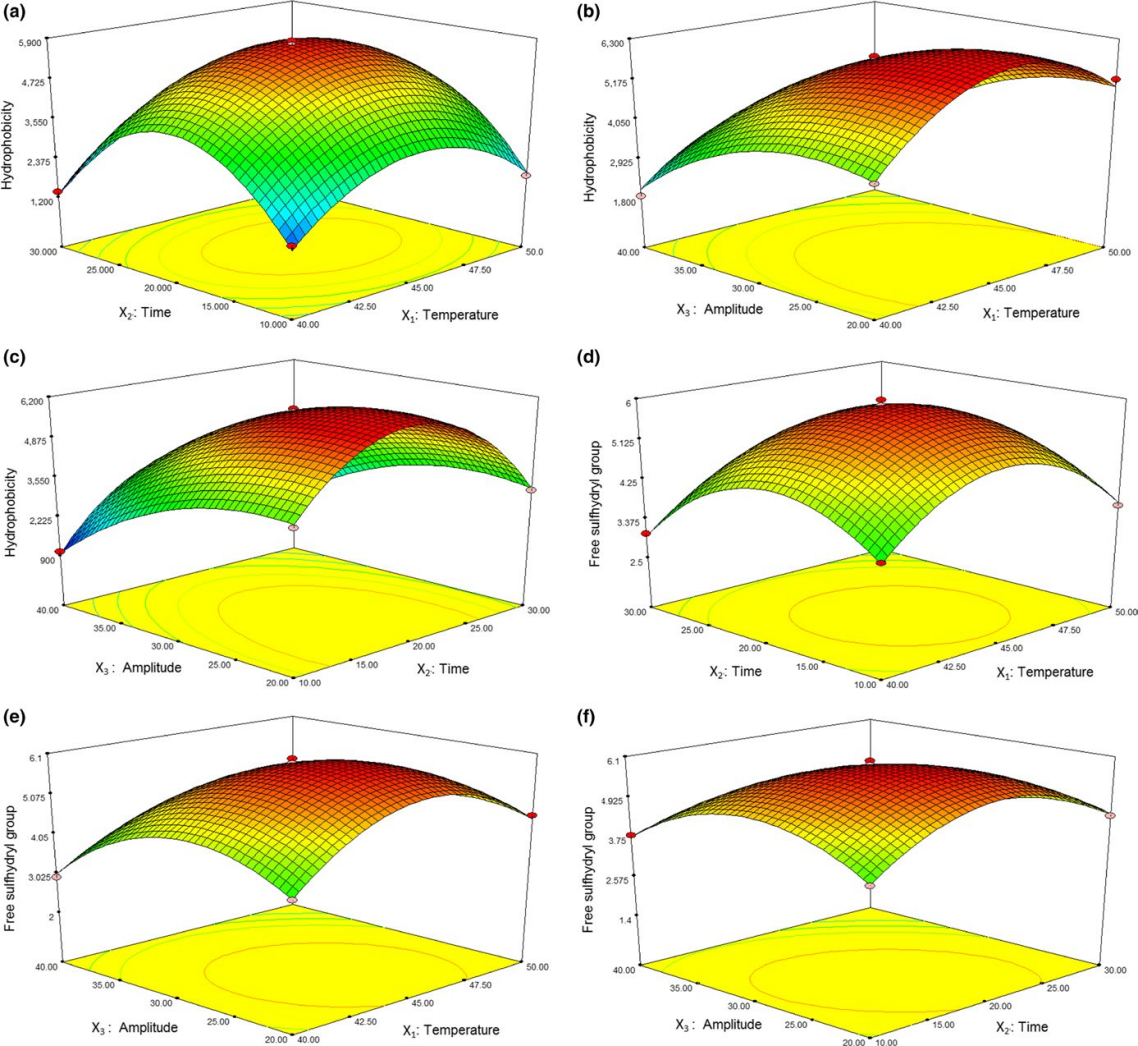
Response surface plot showing effect of three factors and their interactions on the surface hydrophobicity and free sulfhydryl group

Figure 2a–c shows the effect of three factors and their interactions on the surface hydrophobicity. The results indicated that the surface hydrophobicity of ultrasound‐treated β‐lactoglobulin was changed from 1,037.5 to 5,812.08 under different conditions. The interaction between temperature and amplitude affected the surface hydrophobicity significantly (Figure 2b). The maximum surface hydrophobicity of β‐lactoglobulin was 5,812.08 (45°C, 20 min, AP 30%), while the predict value of the surface hydrophobicity was 5,774.40. β‐Lactoglobulin contains a high proportion of hydrophobic amino acid chains, preferentially turned toward the inside of the molecule. The surface hydrophobicity of β‐lactoglobulin was expected to increase when the molecule unfolds during ultrasound treatment. Similar results were reported by Chandrapala, Oliyer, et al. (2012) and Chandrapala, Zisu, et al. (2012).

The mathematical model, representing the effect of three factors on the response values of free sulfhydryl group, can be described by the following quadratic equations (7) with analysis of variance shown in Table 3.

(7) $$\begin{array}{cc} Y_{2}=\; & 5.88-0.11X_{1}-0.49X_{2}-0.83X_{3}-0.12X_{1}X_{2}-0.39X_{1}X_{3}\\ & -0.69X_{2}X_{3}-1.35X_{1}^{2}-1.28X_{2}^{2}-1.18X_{3}^{2} \end{array}$$

**Table 3:** Variance analysis of free sulfhydryl group

Source	Sum of squares	*df*	Mean square	*F*‐value	*p*‐Value Prob > *F*
Model	25.67	9	2.85	320.12	<.0001
*X*_1_‐Temperature	0.09	1	0.09	9.90	.0255
*X*_2_‐Time	1.91	1	1.91	214.52	<.0001
*X*_3_‐Amplitude	5.46	1	5.46	613.08	<.0001
*X*_1_ *X*_2_	0.06	1	0.06	6.20	.0552
*X*_1_ *X*_3_	0.62	1	0.62	69.17	.0004
*X*_2_ *X*_3_	1.93	1	1.93	216.89	<.0001
*X*_1_^2^	6.74	1	6.74	756.32	<.0001
*X*_2_^2^	6.08	1	6.08	682.62	<.0001
*X*_3_^2^	5.17	1	5.17	580.38	<.0001
Residual	0.04	5	0.01		
Lack of fit	0.03	3	0.01	1.47	.4285
Pure error	0.01	2	0.01		
Cor total	25.71	14			

Comment: *R* ^2^ = .9983, Adjusted *R* ^2^ = .9851, Predicted *R* ^2^ = .9797, Adequate Precision = 58.09.

*Y*_2_ is the free sulfhydryl group, *X*_1_ *, X*_2_, and *X*_3_ are the coded variables for temperature, time, and amplitude, respectively. Table 3 shows that the *p*‐value of the response model was significant (*p *≤* *.0001), but the lack of fit was insignificant (*p *=* *.4285 > .1), indicating that the model could be used to analyze and predict the response of free sulfhydryl group. The *X*_1_ *, X*_2_ *, X*_3_ *, X*_1_ *X*_3_ *, X*_2_ *X*_3_ were significant in model terms.

Figure 2d–f shows the effect of three factors and their interactions on the free sulfhydryl group. The interaction between temperature and amplitude (Figure 2e) and the interaction between time and amplitude (Figure 2f) affected the free sulfhydryl group significantly (*p *<* *.05). At 20 min, within the range of temperature applied in the experiment, free sulfhydryl group first increased and then decreased with the increase in amplitude. Similarly, up to 45°C, the free sulfhydryl group increased slightly in the early stage, and the trend was reversed with the increase in amplitude. The results indicated that the free sulfhydryl group of ultrasound‐treated β‐lactoglobulin was changed from 1.42 to 5.97 μmol/g under different conditions. The maximum free sulfhydryl group of β‐lactoglobulin was 5.97 μmol/g (45°C—20 min—AP 30%), while the predicted value of the free sulfhydryl group was 5.88 μmol/g. Ultrasound treatment increased the free sulfhydryl group of β‐lactoglobulin, which might be due to the cavitation during ultrasound treatment.

**Figure 3:**
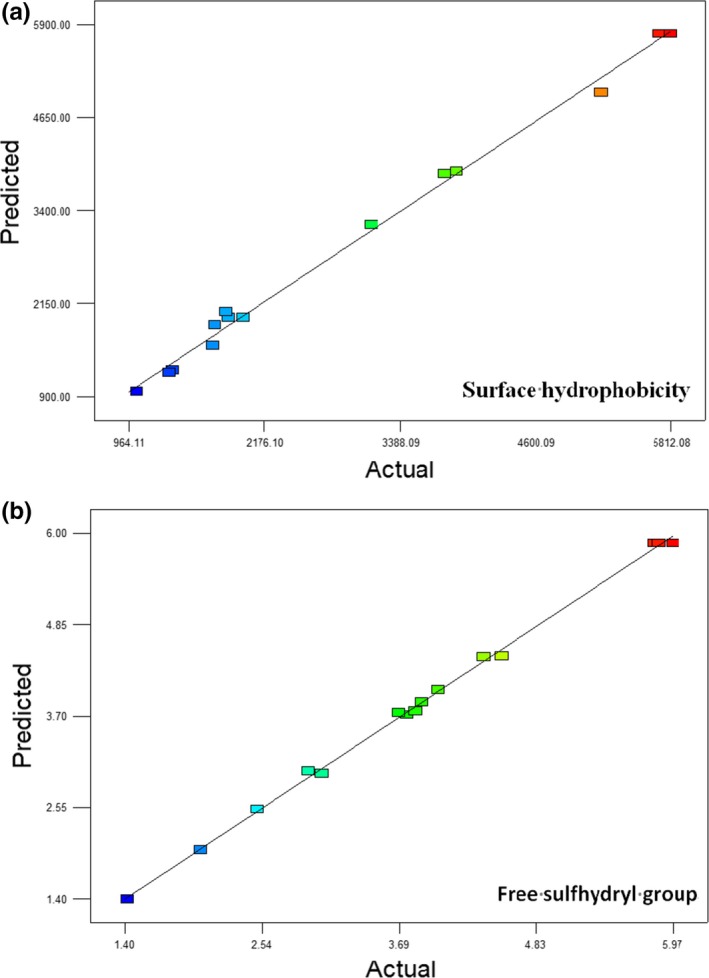
Comparison between actual values and predicted values

**Figure 4:**
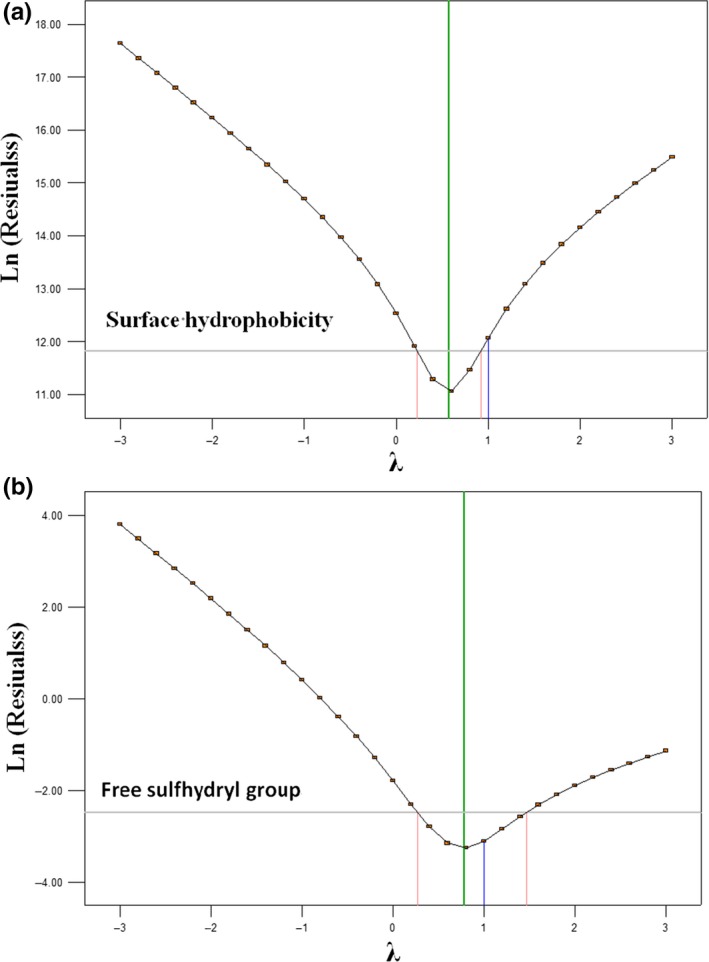
Residual plots

The predicted results of surface hydrophobicity and free sulfhydryl group were close to the observed experimental responses according to the models (Figure 3). The residual plots for power transforms are shown in Figure 4, and the experimental data were in the confidence interval with no abnormal data. So the optimized conditions of ultrasound parameter treated on β‐lactoglobulin were to be 45°C, 20 min, and amplitude of 30%.

### 3.3 Particle size and size distribution of β‐lactoglobulin

**Figure 5:**
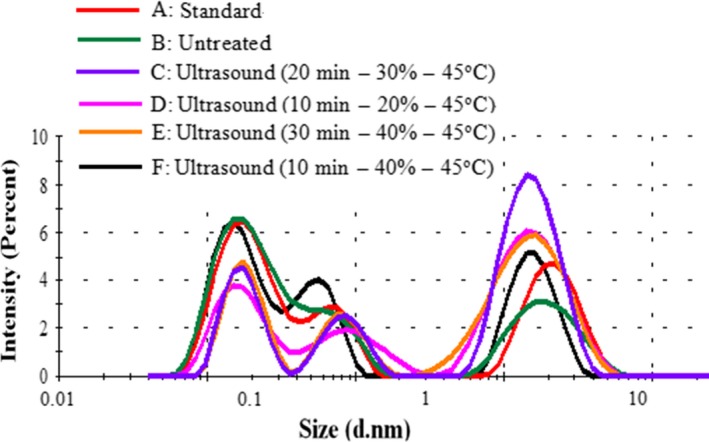
The size distribution of β‐lactoglobulin

Based on the BBD design, the samples of experiment 10, 11, 12, and 13 were selected for further studies. The particle size and PDI of β‐lactoglobulin are shown in Table 4 and Figure 5. The PDI of β‐lactoglobulin was 0.44 ± 0.23, 0.41 ± 0.26, 0.54 ± 0.10, 0.50 ± 0.24, 0.67 ± 0.32, 0.74 ± 0.03, respectively, which was within the range of 0.2 to 0.8. Figure 5 shows that the ultrasonicated samples showed a broader particle distribution compared with the untreated. Similar results were reported by Jambrak et al., 2009 and Hu et al. (2013).

**Table 4:** Particle size and zeta potential of β‐lactoglobulin (1%, ω*/v*)

Sample	Particle size (nm)	PDI	Zeta potential (mV)
A (Standard)	1.21 ± 0.05^a^	0.44 ± 0.23^a^	−18.77 ± 1.79^a^
B (Untreated)	1.26 ± 0.08^a^	0.41 ± 0.26^a^	−15.47 ± 1.60^a^
C (20 min, AP 30%, 45°C)	1.66 ± 0.03^c^	0.54 ± 0.10^b^	−27.63 ± 3.30^c^
D (10 min, AP 20%, 45°C)	1.53 ± 0.15^b^	0.50 ± 0.24^b^	−22.47 ± 2.08^b^
E (30 min, AP 40%, 45°C)	1.61 ± 0.10^c^	0.67 ± 0.32^b^	−25.30 ± 1.61^c^
F (10 min, AP 40%, 45°C)	1.49 ± 0.12^b^	0.74 ± 0.03^c^	−20.16 ± 2.41^b^

Column with different supercase letter means significant difference at *p *<* *.05.

Our results demonstrated that the dispersion system measurement was suitable to determine the particle size and distribution by dynamic light scattering.

The particle sizes of standard and untreated β‐lactoglobulin were 1.21 ± 0.05 and 1.26 ± 0.08 nm, respectively. The particle sizes of ultrasound‐treated samples were from 1.49 ± 0.12 to 1.66 ± 0.03 nm. According to the results, particle size reduction became significant as the amplitude changed from 30% to 40% (*p *<* *.05). While the particle size significantly increased from 1.49 ± 0.12 nm to 1.66 ± 0.03 nm with the increase in sonication time (*p *<* *.05), the particle size decreased to 1.61 ± 0.10 nm at 30 min. The changes in particle size and PDI after ultrasound treatment might be caused by formation of soluble aggregates by the forces of cavitation or small aggregates after ultrasound treatment (Gulseren, Guzey, Bruce, & Weiss, 2007).

### 3.4 Zeta potential of β‐lactoglobulin

The zeta potential of β‐lactoglobulin‐untreated and ultrasound‐treated is shown in Table 4. The absolute zeta potential of ultrasound‐treated β‐lactoglobulin increased significantly from 15.47 ± 1.60 to 27.63 ± 3.30 mV with the increase in amplitude from 20% to 30% (*p *<* *.05), then decreased gradually to 20.16 ± 2.41 mV at the amplitude of 40%. At 10 min, the absolute zeta potential of ultrasound‐treated β‐lactoglobulin decreased from 22.47 ± 2.08 to 20.16 ± 2.41 mV with the increase in amplitude from 20% to 40%. Despite these changes, the zeta potential of β‐lactoglobulin remained negative, which was similar with previous studies reported by Dombrowski et al. (2016). Generally, the zeta potential of the protein solution is negative when there are more negatively charged amino acids than positively charged amino acids. The results indicated that much more negatively charged amino acids than positively charged amino acids were contained in β‐lactoglobulin, and the surface charge characteristics and molecular interactions of β‐lactoglobulin were influenced by ultrasound treatment.

### 3.5 Solubility of β‐lactoglobulin

**Figure 6:**
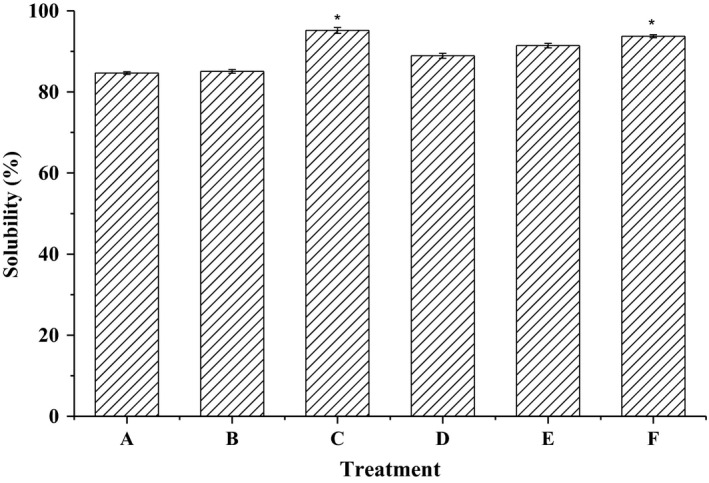
The solubility of β‐lactoglobulin. A: β‐lactoglobulin (standard); B: β‐lactoglobulin (untreated); C: ultrasound‐treated (20 min, AP 30%, −45°C); D: ultrasound‐treated (10 min, AP 20%, 45°C); E: ultrasound‐treated (30 min, AP 40%, 45°C); F: ultrasound‐treated (10 min, AP 40%, 45°C)

The solubility of β‐lactoglobulin increased significantly from 84.66% to 95.17% (*p* < .05) after ultrasound treatment (Figure 6). There was a significant increase in the solubility of β‐lactoglobulin with the increase in amplitude from 20% to 40% at 10 min or 20 min, while the solubility of β‐lactoglobulin decreased at the amplitude of 40% and 30 min. This could be due to a pI shift of ultrasound‐treated β‐lactoglobulin, with a reduction in the number of exposed positive charges and an increase in the net negative charge. Nacka, Chobert, Burova, Le'onil, and Haertle’ (1998) reported that the native β‐lactoglobulin was soluble in the pH range 3–8, with minimum solubility of 80% in the region of its isoelectric point (pI). Chevalier, Chobert, Dalgalarrondo, and Haertle’ (2010) reported that native β‐lactoglobulin (2 mg/ml) was soluble in the pH range 2–10. Ultrasound treatment could change the conformation and structure of β‐lactoglobulin, associated with the surface hydrophobic and hydrophilic interactions. So the solubility of β‐lactoglobulin increased with the hydrophilic amino acid residues reoriented toward water phase.

### 3.6 Chromatographic profiles of β‐lactoglobulin

**Figure 7:**
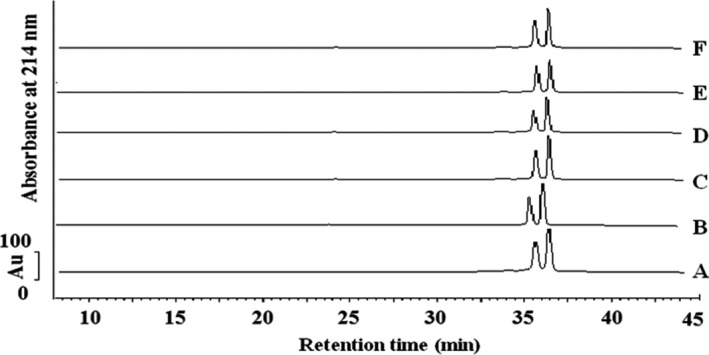
Chromatogram detection of β‐lactoglobulin samples with C_8_ column (5 μm, 300 Å, 4.6 × 250 mm). Peak 1 was β‐lactoglobulin B; Peak 2 was β‐lactoglobulin A. A: β‐Lactoglobulin (standard); B: β‐lactoglobulin (untreated); C: ultrasound‐treated (20 min, AP 30%, 45°C); D: ultrasound‐treated (10 min, AP 20%, 45°C); E: ultrasound‐treated (30 min, AP 40%, 45°C); F: ultrasound‐treated (10 min, AP 40%, 45°C)

An optimal absorption wavelength at 214 nm for β‐lactoglobulin was determined by full spectrum scanning (Bonfatti, Giantin, Rostellato, Dacasto, & Carnier, 2013). The two large peaks during eluting at 35 and 37 min represent β‐lactoglobulin A and B variants, respectively. Similar chromatographic profiles for β‐lactoglobulin were observed previously (Lucena, Alvarez, Menendez, Riera, & Alvarez, 2006). Figure 7 shows HPLC chromatography of untreated and ultrasound‐treated β‐lactoglobulin. There were no significant changes in the peaks of β‐lactoglobulin compared with the native β‐lactoglobulin.

### 3.7 Molecular weight distribution of β‐lactoglobulin

**Figure 8:**
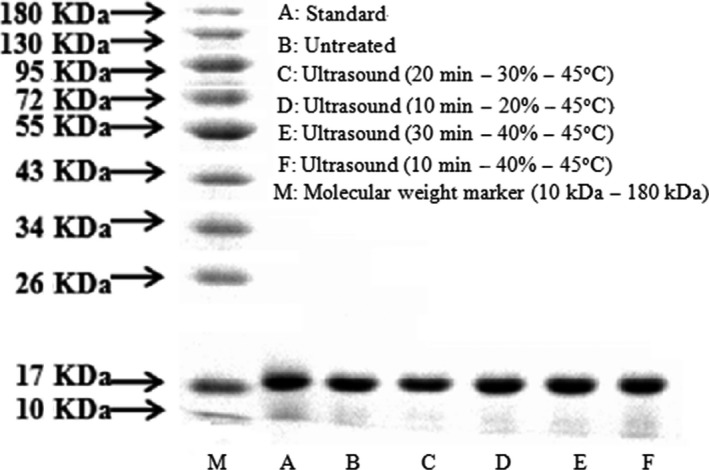
Protein profiles of untreated and ultrasound‐treated β‐lactoglobulin

The protein profiles for untreated (A–B) and ultrasound‐treated (C–F) samples are shown in Figure 8. The separated β‐lactoglobulin (B–F) showed one band at about 18 kDa corresponding to monomeric form, similar to the standard β‐lactoglobulin. As shown in the protein electrophoresis profiles, ultrasound treatment had no significant effect on the primary structure of β‐lactoglobulin. It can be inferred that the aggregation of β‐lactoglobulin may be partially resulted from noncovalent interactions, such as electrostatic and hydrophobic interactions (Arzeni et al., 2012). Similar results had been obtained in the SDS‐PAGE studies of WPC, dairy proteins, and β‐lactoglobulin as reported previously (Chandrapala et al., 2013; O'Sullivan, Arellano, Pichot, & Norton, 2014).

### 3.8 Fourier Transform Infrared (FT‐IR) of β‐lactoglobulin

Specific secondary structures within the protein were associated with particular hydrogen‐bonding patterns. The amide band (1,700–1,600 cm^−1^) was characteristic for the protein having predominant β‐sheet structures and contained most of the information on the secondary structure of protein (Dong et al., 1996). The adsorption at 1,653 cm^−1^ was assigned to α‐helix structures, the adsorption around 1,636 cm^−1^ corresponded to β‐sheet, and the adsorption around 1,645 cm^−1^ was regarded as the random coil structures (Sangho, Lefèvre, Subirade, & Paquin, 2009).

**Figure 9:**
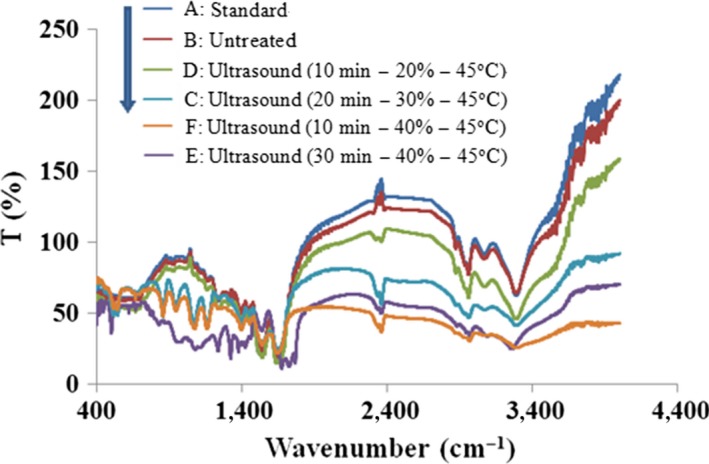
FT‐IR spectrum of untreated and ultrasound‐treated β‐lactoglobulin (1%, ω*/v*)

The FT‐IR spectra of untreated and ultrasound‐treated β‐lactoglobulin solutions are shown in Figure 8. It revealed that β‐lactoglobulin had the significant absorbance in unsaturated C–H (over 3,000 cm^−1^), O–H (3,300–2,500 cm^−1^), –NH_2_ and –NH (3,400–3,100 cm^−1^), =C–H (3,100–3,000 cm^−1^), –CH_3_ (2,960 ± 10 cm^−1^), –C=O (1,850–1,600 cm^−1^), amide band (1,700–1,600 cm^−1^) and C=C(1,680–1,620 cm^−1^), C–N (1,360–1,180 cm^−1^), C–OH (1,300–1,200 cm^−1^), C–O (1,300–1,000 cm^−1^), C=S (1,250–1,000 cm^−1^), C–O–C (1,150–900 cm^−1^), C–C (1,100–1,020 cm^−1^), COO– (780–660 cm^−1^), and C–S (730–600 cm^−1^). There was a significant broadening and shifting of amide band to 1,700–1,600 cm^−1^. Compared with the untreated β‐lactoglobulin, the absorbance of ultrasound‐treated samples decreased gradually, indicating an increase in α‐helix and β‐sheet structures and a decrease in random coil structure of β‐lactoglobulin after ultrasound treatment. The changes in structure of β‐lactoglobulin might be due to the unfolding of the compact structure and the formation of denaturation and aggregation (Striolo, Favaro, Elvassore, Bertucco, & Noto, 2003) (Figure 9).

### 3.9 Changes in fluorescence property of β‐lactoglobulin

**Figure 10:**
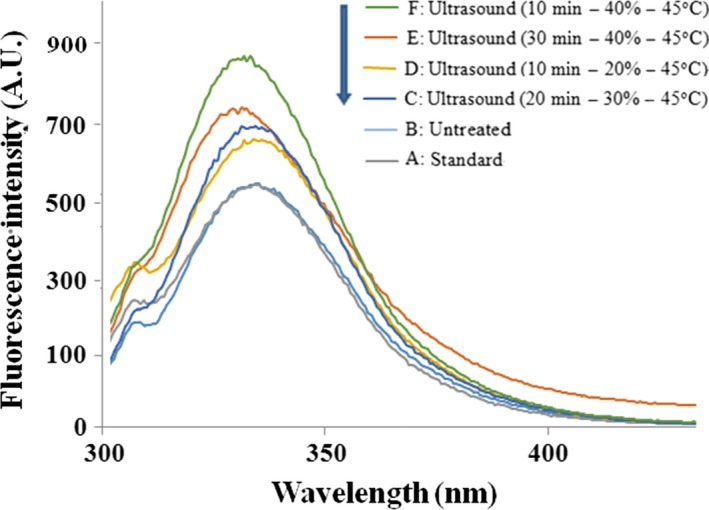
Intrinsic fluorescence emission spectra of untreated and ultrasound‐treated β‐lactoglobulin (0.005%, ω*/v*)

The fluorescence spectrum of untreated and ultrasound‐treated β‐lactoglobulin was observed, and the results are shown in Figure 10. Ultrasound‐treated β‐lactoglobulin contributed to an increase in relative fluorescence intensity. With the amplitude increasing, the relative intensity was increased. A minimum relative fluorescence intensity appeared when the β‐lactoglobulin ultrasound treated at amplitude of 40%. It was probable that β‐lactoglobulin was partially unfolded, and the Trp residues (19Trp and 61Trp) in hydrophobic environment were more or less exposed to the strong hydrophobic environment. When excited at 280 nm, the native β‐lactoglobulin exhibited a maximum fluorescence emission (λ_max_) at 334 nm. Ultrasound‐treated β‐lactoglobulin induced a shift of λ_max_ from 334 to 329 nm. It may be due to the Trp residues moving away from the aqueous phase as a result of the protein conformational changes induced by ultrasound (Stanic‐Vucinic, Prodic, Apostolovic, Nikolic, & Velickovic, 2013).

### 3.10 Changes in UV spectroscopy of β‐lactoglobulin

**Figure 11:**
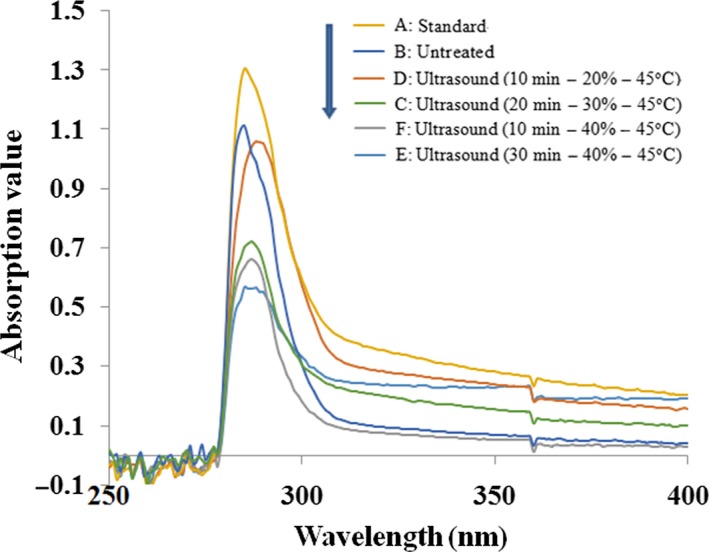
UV spectroscopy of untreated and ultrasound‐treated β‐lactoglobulin (0.05%, ω*/v*)

The UV spectra of untreated and ultrasound‐treated β‐lactoglobulin are shown in Figure 11. Because of the conjugated olefinic bond absorption of tryptophan, tyrosine, and phenylalanine, the maximum absorption peak of untreated β‐lactoglobulin was at 288 nm. Ultrasound‐treated β‐lactoglobulin induced a little shift of λ_max_ from 288 to 285 nm. As shown in Figure 11, ultrasound‐treated β‐lactoglobulin contributed to a decrease in ultraviolet absorption. With the amplitude increasing, the ultraviolet absorption decreased. At amplitude of 40%, the ultraviolet absorbance reached a minimum. It may be due to the changes in the distribution of several amino acids. The result indicated ultrasound treatment changed the structure of β‐lactoglobulin.

## 4 CONCLUSION

Ultrasound treatment had considerable impact on physicochemical properties and structure of β‐lactoglobulin. The maximal surface hydrophobicity and free sulfhydryl of β‐lactoglobulin were 5,812.08 and 5.97 μmol/g, respectively. The particle size, zeta potential, and solubility were significantly increased after ultrasound treatment. Ultrasound treatment can change the structure of β‐lactoglobulin by altering α‐helix and β‐sheet structures. Intrinsic fluorescence intensity of β‐lactoglobulin was increased, but UV absorption of β‐lactoglobulin was decreased after ultrasound treatment. There were no significant changes in high‐performance liquid chromatography and protein electrophoretic patterns. Ultrasound treatment can be used to improve physicochemical properties of β‐lactoglobulin. These studies provide a theoretical basis for the application of surface properties of β‐lactoglobulin.

## CONFLICT OF INTEREST

The authors declare that they have no conflict of interest.
